# Mechanism of Paroxetine (Paxil) Inhibition of the Serotonin Transporter

**DOI:** 10.1038/srep23789

**Published:** 2016-04-01

**Authors:** Bruce A. Davis, Anu Nagarajan, Lucy R. Forrest, Satinder K. Singh

**Affiliations:** 1 Department of Cellular and Molecular Physiology, Yale University School of Medicine, 333 Cedar Street, New Haven, CT 06520 USA; 2 Computational Structural Biology Section, National Institute of Neurological Disorders and Stroke, 35 Convent Drive, Bethesda, MD 20892 USA

## Abstract

The serotonin transporter (SERT) is an integral membrane protein that exploits preexisting sodium-, chloride-, and potassium ion gradients to catalyze the thermodynamically unfavorable movement of synaptic serotonin into the presynaptic neuron. SERT has garnered significant clinical attention partly because it is the target of multiple psychoactive agents, including the antidepressant paroxetine (Paxil), the most potent selective serotonin reuptake inhibitor known. However, the binding site and orientation of paroxetine in SERT remain controversial. To provide molecular insight, we constructed SERT homology models based on the *Drosophila melanogaster* dopamine transporter and docked paroxetine to these models. We tested the predicted binding configurations with a combination of radioligand binding and flux assays on wild-type and mutant SERTs. Our data suggest that the orientation of paroxetine, specifically its fluorophenyl ring, in SERT’s substrate binding site directly depends on this pocket’s charge distribution, and thereby provide an avenue toward understanding and enhancing high-affinity antidepressant activity.

Transmission of nerve impulses across chemical synapses is a fundamental means of communication in the brain and absolutely required for an organism’s survival. An essential component of this process is the reuptake of released neurotransmitters into presynaptic neurons and glia by sodium-dependent neurotransmitter transporters^1^. One of the most clinically and pharmacologically significant of these proteins is the serotonin (5-hydroxytryptamine, 5-HT) transporter (SERT), a member of the neurotransmitter:sodium symporter family (NSS)^2^. SERT dysfunction has been implicated in multiple neuropsychiatric diseases such as depression^3^,^4^, generalized anxiety^4^, autism^5^,^6^, and obsessive-compulsive disorder (OCD)^7^,^8^. It is also the target of psychoactive agents such as the therapeutic tricyclic antidepressants (TCAs) and selective serotonin reuptake inhibitors (SSRIs) as well as the addictive cocaine and amphetamine derivative “ecstasy”^9^,^10^.

Such clinical importance has stimulated intense scrutiny into the location of antagonist binding sites and the molecular mechanism of inhibition, with the long-range objective of designing more effective therapeutics with fewer side effects. In addition to seminal work combining cross-species chimeras, site-directed mutagenesis, and the substituted accessibility method with flux and binding assays (reviewed in ref. 2), homology models^11^,^12^,^13^,^14^,^15^ based on the structure of LeuT^16^, a bacterial nonpolar amino acid transporter^17^ and NSS orthologue, have identified transmembrane helices and several residues responsible for various inhibitor potencies and permitted a semi-quantitative evaluation of conformational changes associated with drug binding in SERT. Subsequent studies with LeuT itself have identified two potential drug binding sites, one at the orthosteric substrate binding pocket (S1) and a second (S2) approximately 11–12 Å above S1 in a so-called extracellular vestibule (Fig. 1a)^18^,^19^,^20^, the latter of which has been elegantly pinpointed as the elusive allosteric site in SERT^21^.

The SSRI paroxetine is among the most widely-prescribed and therapeutically effective SERT antagonists approved to treat a wide range of neuropsychiatric ailments, including depression, OCD, panic disorder, social phobia, posttraumatic stress disorder, generalized anxiety disorder, and premenstrual dysphoric disorder^22^,^23^. Paroxetine is also the highest-affinity and one of the most selective SERT inhibitors known, with a dissociation constant (*K*_d_) of <1 nM^22^,^24^,^25^,^26^, an extremely slow off-rate^27^, and a *K*_i_ of ~1 nM for inhibition of 5-HT uptake^24^,^25^. As with many antidepressants, its binding is dependent on sodium but not chloride^11^,^24^, and its kinetic mechanism of substrate transport inhibition is competitive^24^,^25^,^28^. Chemically, paroxetine is a lipophilic, enantiomerically pure, (−)-trans-3,4-disubstituted piperidine derivative with benzodioxol and fluorophenyl substituents (Fig. 1b)^29^.

Decades of functional data indicate that most SSRIs and TCAs occupy the S1 site^2^, partly substantiated by the crystal structures of a nonfunctional dopamine transporter from *Drosophila melanogaster*^30^ as well as of a hybrid of LeuT and the biogenic amine transporters (BATs), dubbed “LeuBAT”^31^. However, equally compelling pharmacological, biochemical, and mutagenesis data suggest that amino acids implicated in high-affinity paroxetine binding may not overlap with those thought to be involved in recognizing other inhibitors or the substrate 5-HT^32^,^33^,^34^. Furthermore, there is evidence that paroxetine can also act as an allosteric modulator of SERT, although not as effectively as the SSRI escitalopram, the S-enantiomer of racemic citalopram^26^,^35^,^36^,^37^.

The structure of a transport-competent SERT bound to paroxetine has not yet been determined, but some clues as to where this potent SSRI may bind can be gleaned from two independent studies. The first is a systematic analysis of “S1-residue” mutants on the potencies of several SSRIs, SNRIs, and TCAs, from which three subsites (A, B, C) were originally pinpointed^14^ (Fig. 1c–e). The second is a crystal structure of paroxetine bound to the transport-deficient LeuBAT-Δ13 (PDB ID 4MM4)^31^, in which thirteen amino acids within 10 Å of the S1 site in LeuT were mutated to their counterparts in human SERT (hsSERT). Based on this model, a modified definition of the three subsites was proposed, the most substantial difference being the definition of subsite C (referred to here as C^W^), which resides closer to the extracellular side of the protein and includes the negatively-charged glutamate gating residue E493 (Fig. 1d,e).

Although paroxetine was observed to bind in the S1 site of LeuBAT-Δ13, with its piperidine, benzodioxol, and fluorophenyl groups occupying subsites A, B, and C^W^, respectively (Fig. 2), and presumably stabilizing an outward-open state^31^, there are two caveats to this structural model. First, the LeuBAT-Δ13 construct cannot transport any substrate, hinting that it may be perpetually frozen in an outward-open conformation. Second, and perhaps more importantly, the construct binds paroxetine poorly, with a *K*_d_ of only ~430 nM, an almost 500-fold lower affinity than that of hsSERT. This low potency suggests that the manner in which LeuBAT-Δ13 binds paroxetine does not reflect its true interaction with SERT and, consequently, that LeuBAT-Δ13 is not an optimal template for understanding SERT pharmacology. Indeed, a comprehensive analysis of the putative fluoxetine binding site indicated that although fluoxetine does likely bind in S1, its orientation in SERT is almost certainly “reversed” from that seen in LeuBAT^38^.

Here, we address the question of paroxetine binding to SERT with a synergistic combination of computational biology, single-residue cross-species mutagenesis, flux assays, and radioligand binding, on three SERT homologues with disparate paroxetine potencies. While our data support the notion that paroxetine binds at S1, they reveal that its orientation in hsSERT differs from that in LeuBAT-Δ13, involving a distinct region of subsite B, and that the incongruity can be traced primarily to the presence of an acidic amino acid in the orthosteric, S1 binding site.

## Results

### SERT homologue selection, homology modeling, and paroxetine docking

To narrow down the residues responsible for paroxetine recognition, we began by searching for SERT homologues with divergent potencies for paroxetine compared with hsSERT and chose the ones from *Drosophila melanogaster* (dmSERT) and chicken (*Gallus gallus*, ggSERT) (Fig. 3). The published paroxetine *K*_i_ values for inhibition of [^3^H]5-HT uptake are 0.25, 3.4, and 397 nM for hsSERT^39^, dmSERT^40^,^41^, and ggSERT^42^, respectively, although all three homologues transport [^3^H]5-HT with similar Michaelis constants (*K*_m_)^39^,^40^,^41^,^42^,^43^. We first sought to confirm these data in our experimental setup (see Methods) and did indeed observe commensurate steady-state kinetic parameters (Supplementary Table S1) as well as paroxetine potencies (Table 1). Specifically, for wild-type (WT) hs, dm, and ggSERT, the *K*_m_ values were 740, 1,820, and 420 nM (Supplementary Table S1 and Supplementary Figs. S2a, S3a and S4a), respectively, while the corresponding *K*_i_ values were ~2, ~6, and ~60 nM (Fig. 4a and Table 1). Although the absolute *K*_i_ values deviate from those published, the rank order for paroxetine potency remains the same.

To probe the molecular basis for the variable paroxetine potencies and elucidate which of the non-identical amino acid(s) (Fig. 3) might be responsible, we next generated a homology model of each of the SERT homologues based on the crystal structure of the *Drosophila melanogaster* dopamine transporter (dmDAT) complexed with cocaine (PDB ID 4XP4)^44^ (see Methods) and then docked paroxetine into the S1 site (Fig. 5). We did not attempt to dock this SSRI in the S2 site for two reasons. First, previously published studies had shown that paroxetine docked within this crevice in a LeuT-based hsSERT homology model did not prefer any particular orientation^11^. Second, Sorensen *et al.* had convincingly demonstrated that at least one amino acid within each of their designated subsites in the S1 pocket led to a statistically significant loss of paroxetine potency when mutated^14^. To validate the docking protocol, we applied the same algorithm to the LeuBAT-Δ13 construct and were able to recapitulate the crystal structure with a root-mean-square deviation (RMSD) of only 1.3 ± 0.4 Å (Fig. 2).

In these docked poses, as well as in the crystal structure (Fig. 2), the paroxetine binding site in LeuBAT comprises the following residues (with the equivalent hsSERT residues in parentheses): Y21 (Y95), A22 (A96), D24 (D98), P101 (A169), V104 (I172), A105 (A173), Y107 (Y175), Y108 (Y176), F253 (F335), F259 (F341), S355 (S438), G359 (G442), D404 (E493), T408 (T497), and V412 (V501) (Fig. 2). The polar benzodioxol group of paroxetine points into a shallow indentation defined by P101, A105, G359, and S356 (which Wang *et al.*^31^ refer to as “part of subsite B”) while the fluorophenyl points upward into and partially occupies the extracellular vestibule, interacting with Y107, F253, T408, and presumably D404^31^, and which Wang *et al.*^31^ denoted as “subsite C”. Strikingly, the electronegative aryl fluorine^45^ is only 3 Å away from the negatively-charged gating aspartate (D404) (Fig. 2), which may partially explain why the affinity of LeuBAT for paroxetine is so low. In hsSERT this amino acid is a glutamate, E493, one methyl group longer than the aspartate, and the adjacent residue in transmembrane helix (TM) 10 is also a glutamate (E494) (Figs. 3 and 5a), whereas in LeuBAT, it is a hydrophobic phenylalanine (F405) (Figs. 2 and 3). Together, E493 and E494 in hsSERT impose a negative electrostatic potential that probably increases the energetic cost of the fluorophenyl pointing toward the vestibule. Not surprisingly, in the hsSERT-paroxetine model, the fluorophenyl is flipped downward into subsite B between TMs 3 and 8, nestled deeper in the protein, and directed toward residues A169 and I172 (P101 and V104 in LeuBAT) (Fig. 5a). Notably, this alternate paroxetine orientation predicted for hsSERT is still compatible with a salt bridge between the protonated^46^ piperidine ring of paroxetine and the negatively-charged aspartate at position 98, an established feature between amine-containing ligands and all monoamine transporters, including SERT^11^,^12^,^47^,^48^,^49^.

### Functional characterization of SERT homologues implies a vital role for position 169 in paroxetine recognition by hsSERT

**Figure 1:**
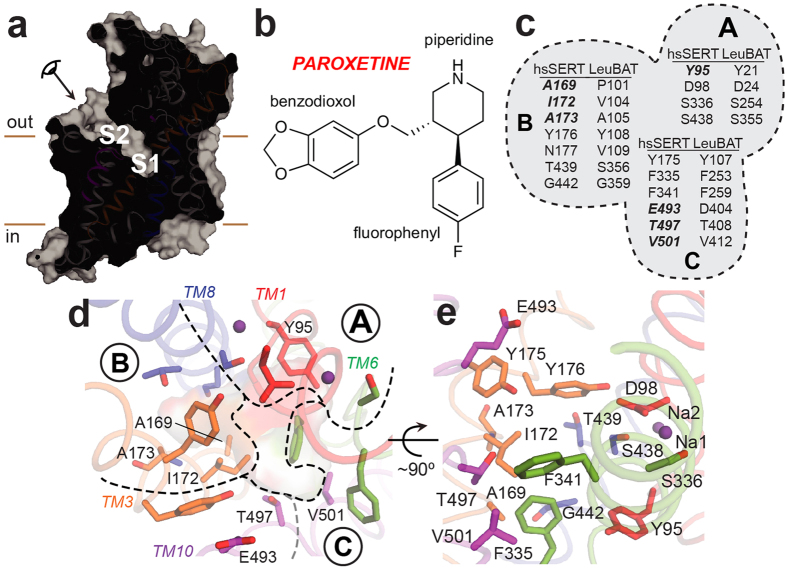
hsSERT substrate binding site and paroxetine chemical structure. (**a**,**d**,**e**) hsSERT homology model in an outward-open state based on the dmDAT-cocaine complex (PDB ID 4XP4). (**a**) Side view of the solvent-accessible surface, with the S1 and S2 ligand-binding sites indicated. The protein is portrayed with cartoon helices colored orange (TM3), blue (TM8), magenta (TM10), and gray (all others). TMs 1 and 6 are foremost but not shown for clarity. Approximate membrane boundaries are represented as brown lines. (**b**) Chemical structure of paroxetine: ((3*S*,4*R*)-3-[(2*H*-1,3-benzodioxol-5-yloxy)methyl]-4-(4-fluorophenyl)piperidine). This SSRI is composed of three functional moieties: piperidine, benzodioxol, and fluorophenyl rings. (**c–e**) Residues composing the S1 binding site. (**c**) Schematic of the subsite definitions for both hsSERT and LeuBAT. The equivalent residues in ggSERT are Y135, D138, S376, and S478 (in subsite A); D209, V212, A213, Y216, N217, T479, and G482 (in subsite B); and Y215, F375, F381, E533, T537, and V541 (in subsite C). The corresponding residues in dmSERT are F90, D93, S328, and S429 (in subsite A); D164, M167, G168, Y171, N172, T430, and G433 (in subsite B); and Y170, F327, F333, N484, P488, and I492 (in subsite C). Residues mutated in this study are in bold italics. (**d,e**) Structural model of the S1 crevice. (**d**) View from extracellular pathway, as indicated in (**a**). The three subsites, A, B, and C, are separated by black dashed lines. Subsite C encompasses definitions from both Sorensen *et al.*^14^ and Wang *et al.*^31^, in which residues implicated only in the LeuBAT structure are to the left of the gray dashed line, referred to here as C^W^. Amino acids mutated in this study are labeled. Cartoon helices are colored as in (**a**), with TMs 1 and 6 colored red and green, respectively. Key groups are shown as sticks and sodium ions as spheres. (**e**) Same representation as (**d**), but viewed from the plane of the membrane. All residues that have been implicated in antagonist recognition within the hsSERT S1 pocket are displayed as sticks and labeled.

Comparison of the three SERT sequences reveals that, within ~6–7 Å of the putative paroxetine site, seven amino acids differ between hsSERT and dmSERT, while only two differ between hsSERT and ggSERT (Fig. 3) despite the latter’s lower potency for paroxetine. Intriguingly, the two positions that diverge in both dmSERT and ggSERT are close to the negatively-charged fluorophenyl moiety of paroxetine docked into the hsSERT model (Fig. 5a). These residues, A169 and I172 (D164 and M167 in dmSERT and D209 and V212 in ggSERT) in subsite B (Figs. 1c,d and 5a) may therefore be responsible for the majority of the differences in potency. Indeed, a previous study implicated both of these residues in paroxetine recognition by ggSERT^42^. To test whether these two positions underlie high affinity in hsSERT, we generated six single-residue “cross-species” mutants: two in hsSERT (A169D and I172M), two in dmSERT (D164A and M167I), and two in ggSERT (D209A and V212I). We then examined paroxetine inhibitory potencies on [^3^H]5-HT transport (see Methods) for all six constructs in transiently-transfected T-REx-293 cells. As shown in Fig. 4b–g and Table 1, we observed a reciprocal paroxetine potency shift for both sets of residues, although the reciprocity was more pronounced for the A-to-D (hsSERT) and D-to-A (dmSERT and ggSERT) mutations (Fig. 4e–g) than for the more conservative I-to-M (hsSERT), M-to-I (dmSERT), or V-to-I (ggSERT) substitutions (Fig. 4b–d). Specifically, we observed a 7-fold decrease in paroxetine potency for hsSERT-A169D and, conversely, a 6–7-fold increase for both dmSERT-D164A and ggSERT-D209A. In fact, paroxetine potency for dmSERT-D164A was more than 2-fold greater than even that for hsSERT-WT (*K*_i_ = 0.9 vs. 2 nM) (Table 1). By contrast, we observed only a 2–3 fold change for hsSERT-I172M (decreased potency), dmSERT-M167I (increased potency), and ggSERT-V212I (increased potency). Interestingly, a previous study of dmSERT, which examined only the role of M167, but not that of D164, also reported a 2.1-fold decrease and 2.6-fold increase in paroxetine potencies for hsSERT-I172M and dmSERT-M167I, respectively, but given the 30–1000-fold potency change discerned for other inhibitors like fluoxetine, sertraline, clomipramine, and citalopram, the authors understandably concluded that such a comparatively modest 2–3 fold reciprocal effect meant that paroxetine likely does not bind in the S1 site but may instead act allosterically^34^.

The observation that the most prominent difference in paroxetine potencies was achieved by swapping an aliphatic residue with a negatively-charged one, and the fact that the aryl fluorine is negatively-charged^45^, are consistent with the predicted paroxetine pose in the homology model of hsSERT-WT (Fig. 5a). Specifically, the fluorine points toward A169 in hsSERT-WT such that introduction of a negative charge at this position is likely to result in direct electrostatic repulsion, consistent with the elevated paroxetine *K*_i_ values exhibited by hsSERT-A169D, dmSERT-WT, and ggSERT-WT. If the effect is indeed electrostatic, then substituting the alanine with a glutamate should exert a similar effect. To test this hypothesis we generated the hsSERT-A169E mutant, but because its turnover rate was barely detectable, we had to assess paroxetine potency via inhibition of [^125^I]RTI-55 binding rather than inhibition of [^3^H]5-HT transport. As illustrated in Fig. 4h, affinities of the A169D and A169E mutants for paroxetine were both approximately 30-fold lower than that of the wild-type construct. Somewhat unexpectedly, data from inhibition of [^125^I]RTI-55 binding revealed a much more profound reduction in paroxetine potencies than that indicated by inhibition of [^3^H]5-HT uptake (30- vs. 7-fold). Nevertheless, such a discrepancy between transport and binding has been noted previously^42^,^50^,^51^ and may reflect the conformationally dynamic nature of transport, with the return step hypothesized to be rate-limiting^52^,^53^,^54^, whereas binding is a more direct reflection of drug-protein interactions.

To examine how electrostatic repulsion (and potentially also steric hindrance) due to the aspartate sidechain at position 169 might alter the affinity of hsSERT for paroxetine, we constructed homology models of both ggSERT (Fig. 5c) as well as dmSERT (Fig. 5d) and then docked paroxetine into these models. According to the ligand poses in the most populated cluster, the salt bridge between the piperidine amine and the aspartate at position 98 was recapitulated in all three homologues, with the piperidine occupying subsite A. However, the fluorophenyl group was no longer directed toward the positions equivalent to hsSERT-A169 and -I172 in subsite B, but was instead pointing into subsite C (Fig. 5c,d). These differences could, of course, reflect other substitutions in these regions of dmSERT and ggSERT (Fig. 3). For example, ggSERT also has a valine at position 172 while dmSERT has a methionine, both of which, as mentioned above, manifest reciprocal potency effects when mutated to the corresponding hsSERT residues, i.e. alanine and isoleucine. In our ggSERT-paroxetine model, the piperidine and benzodioxol moieties occupy subsites A and B, respectively (Fig. 5c), akin to their positions predicted for hsSERT (Fig. 5a). The positioning of the benzodioxol group occurs despite the replacement of I172 in hsSERT with V212 in ggSERT. As mentioned above, the V212I mutation in ggSERT results in a 2–3-fold increase in paroxetine potency (Fig. 4d and Table 1), intimating that the benzodioxol might interact slightly more favorably with the marginally larger isoleucine side-chain (Fig. 5c). By contrast, in dmSERT, the long side-chain of a methionine at this position may prevent the benzodioxol group from occupying subsite B and instead propel it to flip upward toward subsite C (Fig. 5d). An additional complexity with dmSERT is the fact that there are five other non-identical amino acids within 6–7 Å of the putative paroxetine binding site. For instance, its extracellular gating residue is a polar asparagine (N484) rather than an acidic glutamate, and the side chain amide is within hydrogen-bonding distance of one of the oxygen atoms in paroxetine’s benzodioxol ring (Fig. 5d).

**Figure 2:**
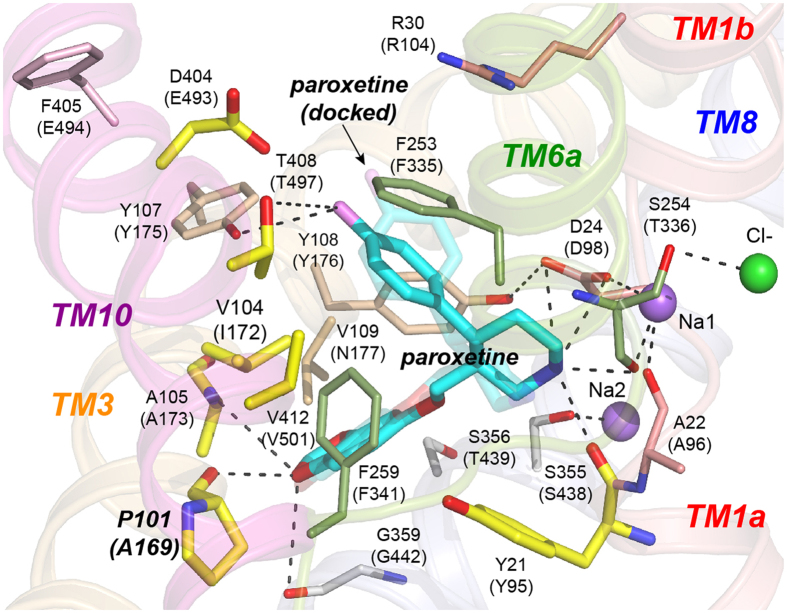
Structure of LeuBAT complexed with paroxetine. For clarity, only TMs 1 (salmon), 3 (orange), 6 (green), 8 (blue), and 10 (magenta) are shown. Amino acids residing in the S1 pocket are illustrated as thin sticks, with their nitrogen and oxygen atoms colored blue and red, respectively, and their carbon atoms colored the same as the helix to which they belong. They are labeled, with the corresponding residues in hsSERT in parentheses. LeuBAT residues whose SERT counterparts were mutated in this study are displayed as thicker sticks with yellow carbon atoms. The carbon atoms of bound and docked paroxetine are cyan, and the fluorine in the fluorophenyl moiety is violet. The best pose of paroxetine docked to LeuBAT is partially transparent. Sodium and chloride ions are depicted as purple and green spheres, respectively. Hydrogen bonds are indicated as black dashed lines.

Cognizant of these complicating factors, we consequently built a second model of hsSERT in which only the alanine at position 169 was replaced by an aspartate. Remarkably, this single change was sufficient to result in different poses for the largest cluster, displacing the fluorophenyl group farther to the extracellular side of subsite C (Fig. 5b), while maintaining the other interactions present in hsSERT-WT (Fig. 5a). Note that in none of the SERT models does the fluorophenyl moiety occupy the C^W^ subsite as it does in LeuBAT (Fig. 2), possibly due to the additional charge repulsion (two carboxyls in hsSERT and ggSERT) and/or hydrophobicity (valine in dmSERT) at the positions equivalent to D404 and F405 of LeuBAT.

Taken together, these biochemical and computational data strengthen our hypothesis that position 169 is crucial for paroxetine binding and dictates its affinity as well as specificity in SERT homologues.

### Effect of other dmSERT mutations on paroxetine potency

**Figure 3:**
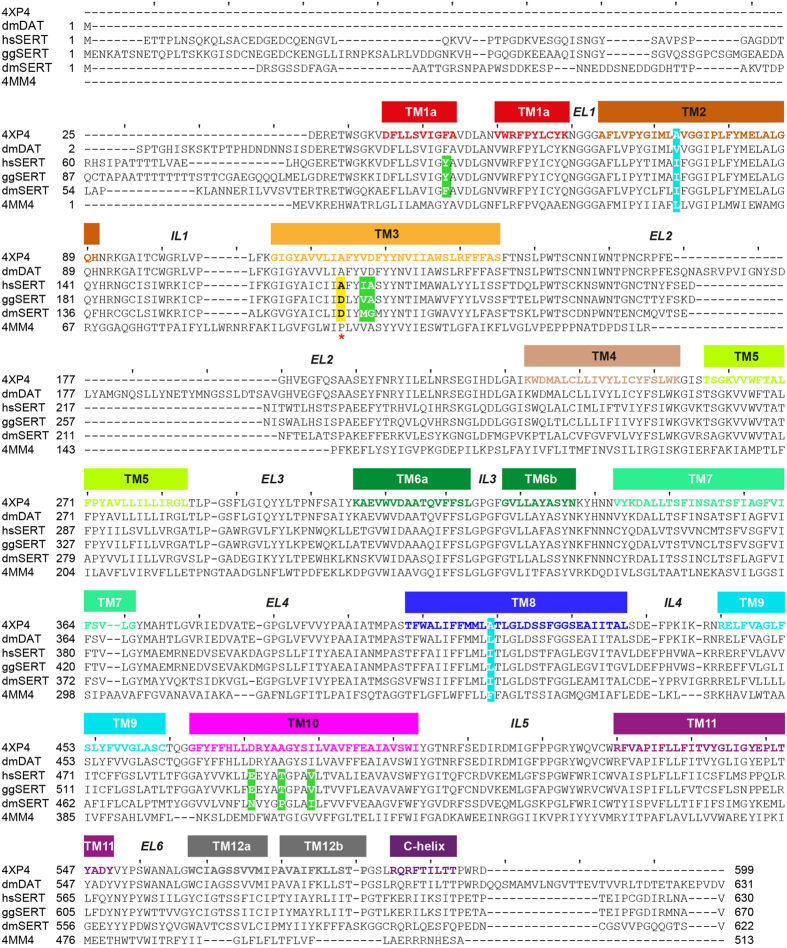
Structure-based amino acid sequence alignment. SERT amino acid sequences aligned with those of the dmDAT-mfc-cocaine complex (PDB ID 4XP4), wild-type dmDAT, and LeuBAT-Δ13 (PDB ID 4MM4). Transmembrane helices are indicated. For clarity, tick marks have been drawn every 10 amino acids. Residues that differ between hsSERT and either dmSERT and/or ggSERT within the S1 site are highlighted in green. The position that exhibits the maximum reciprocal effect on paroxetine potency upon exchange (A169 in hsSERT, D209 in ggSERT, and D164 in dmSERT) is highlighted in yellow with a red asterisk immediately below the position. Residues mutated to Ala in dmDAT to improve thermostability while retaining residual transport activity^44^ are highlighted in cyan.

As mentioned above, hsSERT differs from dmSERT in five other positions around the ligand, aside from A169 (D164 in dmSERT) and I172 (M167 in dmSERT) (Fig. 3), which could also potentially impact paroxetine potency. To exclude this possibility, we substituted individual positions in hsSERT with the equivalent residues in dmSERT, and vice versa, and measured the effect on the paroxetine *K*_i_ for inhibition of [^3^H]5-HT transport. None of these five mutant pairs exhibited reciprocal effects on paroxetine potencies. Instead, they a) did not appreciably alter the *K*_i_ for either mutant (Y95F [F90Y in dmSERT]; Supplementary Fig. S1a,b and Table 1); b) shifted both *K*_i_ values in the same direction (A173G [G168A in dmSERT] and E493N [N484E in dmSERT]; Supplementary Fig. S1c–f and Table 1); or c) altered the *K*_i_ for only one of the two mutants in the pair (T497P [P488T in dmSERT] and V501I [I492V in dmSERT]; Supplementary Fig. S1g–j and Table 1).

The fact that the Y95F (F90Y in dmSERT) exchange barely affected paroxetine potency was surprising since the Y95A replacement in hsSERT perturbs paroxetine potency by more than 70-fold^14^. The Y95F/F90Y result therefore indicates that removal of the hydroxyl is not paramount to removal of the aromatic ring. Thus, Y95 (F90 in dmSERT) may form a π–π or hydrophobic interaction with paroxetine, and according to our model, this interaction possibly involves the fluorophenyl of paroxetine (Fig. 5a). Although the hsSERT-Y95F mutation does not diminish paroxetine potency, it curiously does induce a reciprocal exchange in the potencies for both mazindol (increase) and the SSRI citalopram (decrease)^41^. Moreover, Y95F simultaneously confers the ability on hsSERT to discriminate between citalopram’s R- and S-enantiomers^34^. Although Barker *et al.* were unable to detect appreciable [^3^H]5-HT uptake activity in dmSERT-F90Y and thus could not ascertain if there was a mutually opposite effect on mazindol and citalopram potencies^41^, they did indirectly test the effect of the F90Y mutation by mutating the equivalent position in the human norepinephrine transporter (hsNET), i.e. generating hsNET-F72Y, and measuring inhibition of [^3^H]dopamine transport. These data did indeed demonstrate a reciprocal effect on mazindol and citalopram potencies. We currently cannot explain the disparity with our ability to detect robust [^3^H]5-HT transport activity of the dmSERT-F90Y mutant (Supplementary Fig. S3b, Supplementary Table S1). Nevertheless, our results suggest that the hydroxyl group of Y95 in hsSERT is not critical for inhibition of [^3^H]5-HT transport by paroxetine, in contrast to its reported roles in inhibition by both mazindol and citalopram.

## Discussion

Paroxetine is the most potent SERT inhibitor and one of the most effective therapeutics currently available for a broad spectrum of neuropsychiatric illnesses, yet its precise molecular interactions within its binding site have remained elusive. Part of this dearth of knowledge stems from the paucity of studies expressly targeted toward paroxetine, unlike the prototypical antidepressants escitalopram^13^,^15^,^55^,^56^ or imipramine^12^,^55^, but much of the deficiency is simply due to the unfortunate outcome of not having identified an amino acid which, when mutated, influences the affinity of paroxetine as much as it does that of other antidepressants. One conceivable exception is a report that characterized a series of cross-species chimeras between ggSERT and hsSERT followed by selected site-directed mutants and conjectured that positions 169 as well as 172 in hsSERT play important roles in “sensing the N-methylation state of SERT antagonists”^42^.

Prior investigations into the delineation of antagonist binding sites in SERT have relied on both cross-species comparisons^57^ or, since the arrival of the first LeuT structures^16^,^17^, docking of drugs into reliable homology models^2^,^58^. Here we have integrated the strengths of the two techniques to interrogate the atomic origins of paroxetine’s specificity and extraordinarily high affinity.

**Figure 4:**
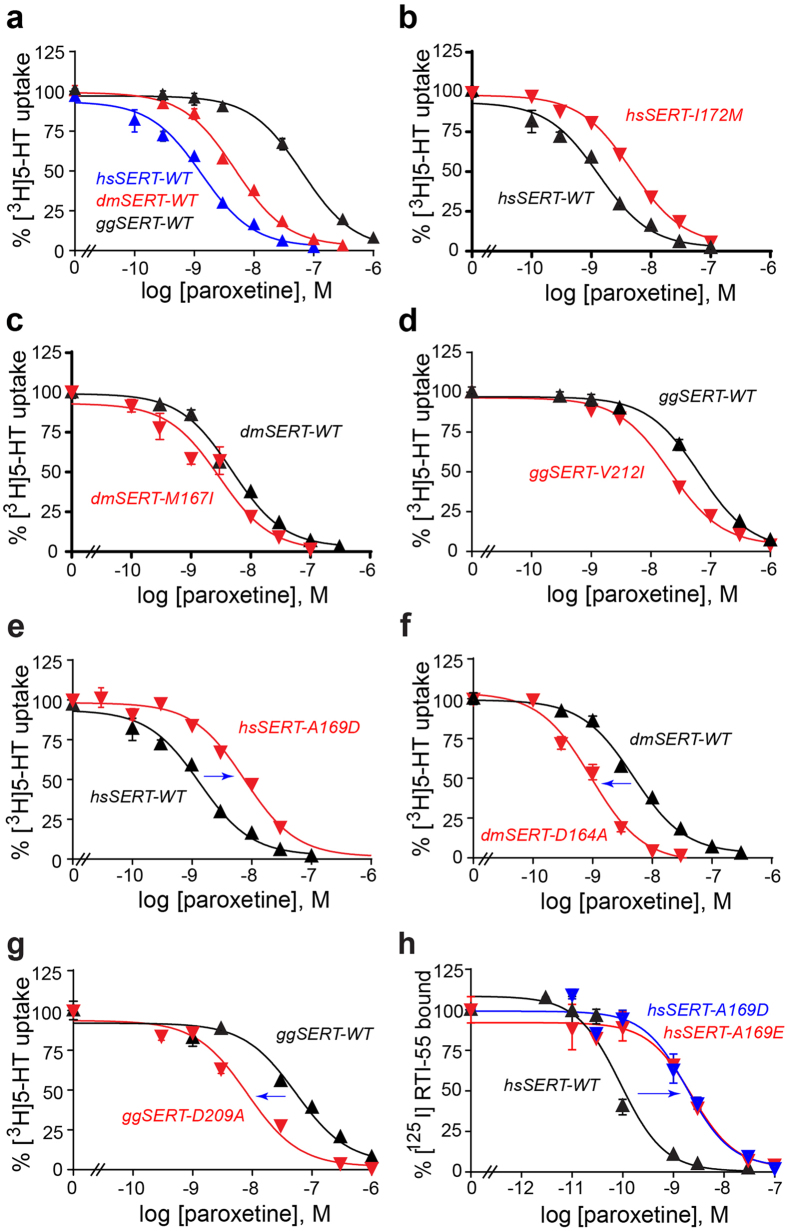
Potencies of SERT homologues & mutants for paroxetine, as assessed by (**a–g**) inhibition of 20 nM [^3^H]5-HT transport in transiently-transfected T-REx-293 cells or (**h**) inhibition of 0.2 nM [^125^I]RTI-55 binding to crude membranes prepared from transiently-transfected T-REx-293 cells. (**a**) Paroxetine inhibition of hsSERT-WT (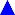), dmSERT-WT (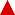), or ggSERT-WT (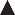). (**b**) Paroxetine inhibition of hsSERT-WT (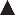) versus hsSERT-I172M (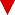). (**c**) Paroxetine inhibition of dmSERT-WT (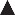) versus dmSERT-M167I (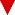). (**d**) Paroxetine inhibition of ggSERT-WT (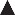) versus ggSERT-V212I (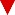). (**e**) Paroxetine inhibition of hsSERT-WT (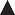) versus hsSERT-A169D (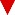). (**f**) Paroxetine inhibition of dmSERT-WT (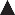) versus dmSERT-D164A (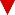). (**g**) Paroxetine inhibition of ggSERT-WT (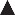) versus ggSERT-D209A (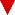). The blue arrows in panels e-g denote a decrease (panel e) or increase (panels f and g) of paroxetine potencies. (**h**) Paroxetine inhibition of [^125^I]RTI-55 binding to hsSERT-WT (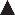), hsSERT-A169D (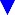), or hsSERT-A169E (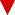). The blue arrow denotes a dramatic decrease in paroxetine binding affinity for both the hsSERT-A169D as well as the –A169E mutants relative to that for hsSERT-WT. The data in each panel represents a typical experiment, each performed in triplicate and repeated a minimum of 3 times (see Table 1). Data points and error bars represent the mean value with the standard error.

**Figure 5:**
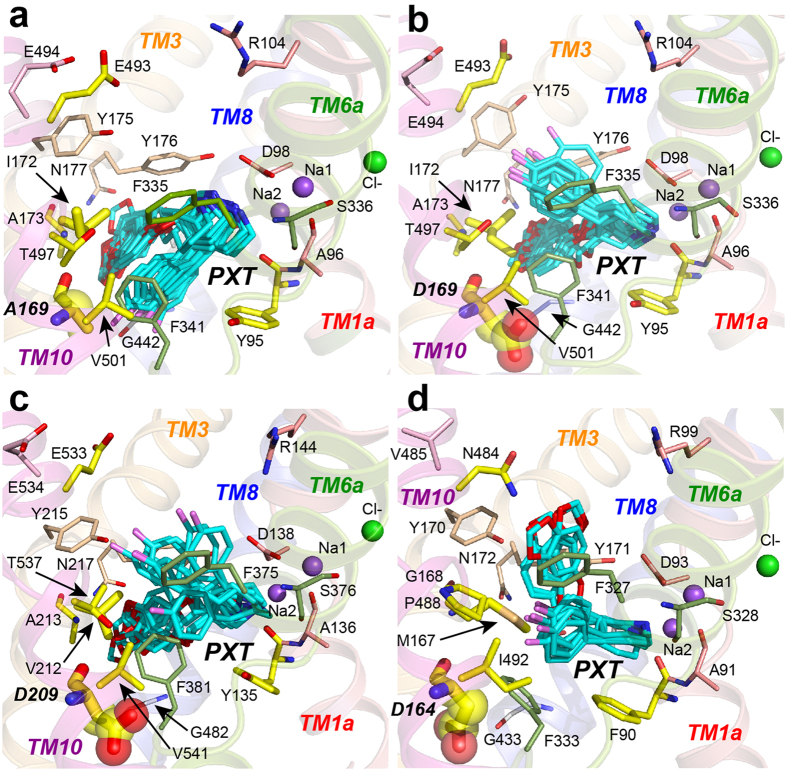
Homology models of SERT homologues and paroxetine (PXT) poses from the largest cluster for (**a**) hsSERT-WT (14 poses), (**b**) hsSERT-A169D (9 poses), (**c**) ggSERT-WT (8 poses), and (**d**) dmSERT (6 poses). Docked paroxetine is colored as in Fig. 2. Transmembrane helices are illustrated as transparent ribbons and colored as follows: TM1 (salmon), TM3 (light orange), TM6 (pale green), TM8 (blue), and TM10 (magenta). For all complexes, oxygen and nitrogen atoms are red and blue, respectively, while carbon atoms are the same color from the helix to which they belong. Carbon atoms from amino acids targeted for mutagenesis, however, are yellow, and these residues are depicted as thicker sticks. Residues depicted as the thickest sticks , with side chain atoms in partially transparent spheres, are those which, when mutated, had the greatest impact on paroxetine potency (A169D, D209A, and D164A in hsSERT, ggSERT, and dmSERT, respectively). Sodium and chloride ions are shown as purple and green spheres, respectively. Note that S438 and its equivalents in dmSERT (S429) and ggSERT (S478) are behind the docked paroxetine models and thus not visible in all panels. Also note that Y216 (equivalent to Y176 in hsSERT and Y171 in dmSERT) is behind the docked paroxetine poses in the ggSERT image (panel **c**).

A peculiar property of the three SERT homologues that we employed in this work is the lack of a direct correlation between paroxetine potency and the degree of sequence identity. Despite the fact that hsSERT shares 82% identity with ggSERT compared with 52% for dmSERT, the paroxetine potency of ggSERT is at least 10-fold lower. This apparent paradox can be reconciled by our data which show that dmSERT possesses at least two “compensatory” residues at positions P488 (T497 in hsSERT) and I492 (V501 in hsSERT) that appear to offset the low identity. For example, introduction of the corresponding hsSERT residue at these positions in dmSERT (P488T and I492V) led to diminished potencies relative to that for dmSERT-WT (Supplementary Fig. S1g–j and Table 1). These compensatory amino acids apparently create an environment in which a single-residue exchange at position 164 (169 in hsSERT) completely switches potencies between the hsSERT and dmSERT homologues. In fact, as mentioned above, this single-residue exchange actually improves dmSERT’s paroxetine potency 2.3-fold beyond that of hsSERT and reduces hsSERT’s paroxetine potency 2.4-fold below that of dmSERT. The docking results indicate that positioning of the benzodioxol group in paroxetine may not be the same in the two proteins (Fig. 5a,d), reflecting the differences in protein environment within subsite C. Nevertheless, the presence of the aspartate or alanine at position 169 leads to 7-fold reciprocal changes in potency and this correlates directly with the position of the fluorophenyl group. Compared with hsSERT-WT (Fig. 5a), the fluorophenyl moiety of paroxetine is predicted to bind to hsSERT-A169D in a less deeply buried orientation (Fig. 5b). Note that the deeper insertion is also not predicted for ggSERT (Fig. 5c) or dmSERT (Fig. 5d), both of which have an aspartate at the equivalent position. Interestingly, while the apparent affinity of ggSERT-D209A for paroxetine improves relative to that of ggSERT-WT, it still falls ~5-fold short relative to that of hsSERT-WT. We speculate that the effect of the I172/V212 substitution (Figs. 3 and 4d) is additive to that of the A169/D209 replacement (Figs. 3 and 4g) potentially accounting for the vestigial change in potency between ggSERT-D209A and hsSERT-WT.

In summary, by employing three, rather than only two, SERT homologues, in combination with computational biology and functional analyses, we have not only confirmed the importance of the previously-identified position 169^42^ and, to a lesser extent, 172, but are also the first to implicate electrostatic contributions in the recognition between a specific functional group of the SSRI paroxetine and a specific SERT position. Although a full SAR study will be required to comprehensively dissect other elements of paroxetine selectivity and especially potency, which is beyond the scope of this work, we have unveiled a pivotal factor in paroxetine-SERT interactions that can now serve as a launching point for future strategic drug development with piperidine derivatives.

## Methods

### Homology modeling

Homology models of hsSERT-WT, hsSERT-A169D, ggSERT, and dmSERT were constructed using the outward-open, cocaine-bound structure of dmDAT (PDB ID 4XP4)^44^ as a template, employing a LeuT-structure-based sequence alignment first presented in Beuming *et al.*^59^. Minor adjustments were made in the loop between TMs 8 and 9 so that the gap was aligned to F454 (hsSERT numbering) rather than W458 (Fig. 3). In addition, residues in EL2 (213–220 of hsSERT; 253–260 of ggSERT; and 208–212 of dmSERT) as well as in EL4 (400–402 of hsSERT; 440–442 of ggSERT; and 390–393 of dmSERT) were modeled without a structural template. These changes to the loop regions improved the quality of their backbone dihedral angles and/or ProQM^60^ scores. The sequence identities between dmDAT (4XP4) and the SERT homologues are 45% (hsSERT-WT and –A169D), 44% (ggSERT), and 46% (dmSERT). The sodium and chloride ions were modeled in their putative sites^61^. For each of the SERT homologues, 2,000 models were generated using MODELLER^62^. The top twenty models were selected according to their agreement with the template’s restraints based on MODELLER’s molpdf score and further analyzed in PROCHECK^63^ to confirm that their backbone dihedral angles remained within the allowed regions of the Ramachandran plot. From this set of twenty, five models with the highest ProQM scores were chosen for each homologue; the RMSD (backbone) spread among these final five models was 1.5, 1.6, and 1.4 Å for hsSERT, ggSERT, and dmSERT, respectively, which helped ensure structural diversity in the subsequent docking step. The average ProQM scores of these models are all excellent: 0.830 ± 0.002, 0.830 ± 0.003, 0.837 ± 0.006, 0.820 ± 0.002 for hsSERT-WT, hsSERT-A169D, ggSERT, and dmSERT, respectively, versus 0.825 for the template 4XP4.

### Docking of Paroxetine

The five models of each homologue were refined in the flexible main chain region between TM6a and TM6b (residues 255–260 of LeuBAT, 337–342 of hsSERT/hsSERT-A169D, 377–382 of ggSERT, and 329–334 of dmSERT) using the Prime loop refinement module v3.4 (Schrödinger) to create more room in the binding site before docking. Paroxetine coordinates were taken from the crystal structure of the nonfunctional LeuBAT-Δ13 structure (PDB ID 4MM4)^31^ and converted to mol format using the online SMILES translator^64^. The LigPrep module (v.2.8) of MacroModel 2013.2 (Schrödinger) was then used to obtain a low energy 3D structure for paroxetine, which was subsequently docked into LeuBAT (as a positive control) and into models of each of the SERT homologues. Docking was performed on the Prime-refined models of each SERT homologue using the InducedFit protocol (Schrödinger)^65^,^66^, after which all poses were pooled together. The docking procedure consisted of three stages: First, four residues in the binding site were converted to alanine to further enlarge the binding site (LeuBAT residues 107, 252, 253, and 259; hsSERT/hsSERT-A169D residues 175, 334, 335, and 341; ggSERT residues 215, 374, 375, and 381; and dmSERT residues 170, 326, 327, and 333). Multiple conformations and orientations of paroxetine in the binding site were docked into this modified site using Glide v6.1 (Schrödinger)^67^,^68^,^69^; the docked poses were screened according to the Standard Precision (SP) scoring function with a “softened” van der Waals potential (scaled down by a factor of 2). Second, the protein structure was refined around these initial docked conformations. Side chains within 5 Å of paroxetine (including the four residues mentioned above) were rebuilt, refined, and energy minimized along with the ligand, using Prime v3.4 (Schrödinger); up to 20 protein-ligand complexes (“poses”) from this step, with energies <30 kcal/mol of the lowest-energy conformation, were retained. Third, the ligand was re-docked into the newly optimized protein structure for the <20 selected poses from stage 2. The poses from all five structures of each protein were pooled together and clustered using the average-linkage clustering program NMRCLUST^70^.

The largest cluster of poses for each protein was examined, and a representative protein-ligand complex for each cluster was chosen based on three criteria: 1) the positions of sodium and chloride ions in the docked structure should remain close to those in the template; 2) the number of hydrogen bonds should be maximized; and 3) the complex should contain as few “bad/ugly” contacts as possible, as defined by the formula *C* = *d*_ij_/(*r*_i_ + *r*_j_), where *d*_ij_ is the distance between atomic centers *i* and *j*, and *r*_*i*_ and *r*_*j*_ are their respective atomic radii. *C* is defined for each atom pair and monotonically increases for each of the contact types, with default cutoff values of *C*(good) = 1.30 Å, *C*(bad) = 0.89 Å and *C*(ugly) = 0.75 Å.

### Molecular biology

cDNA encoding hsSERT and dmSERT (both kindly provided by Randy Blakely) were subcloned into a modified eukaryotic expression vector pcGFP-EU^71^ in which the genes encoding GFP and the C-terminal histidine tag had been deleted (pcNoTag). Mutations in hs and dmSERTs were generated using the Quikchange method and fully sequenced. ggSERT and associated mutants were synthesized by GenScript (Piscaway, NJ) and subcloned into pcNoTag.

### Cell culture for flux assays

Mammalian cells (T-REx-293, Life Technologies) were grown and maintained at 37 °C in 100-mm tissue culture plates in a 5% CO_2_ humidified incubator in Dulbecco’s modified Eagle’s medium (DMEM) containing 10% fetal bovine serum (FBS) (Atlanta Biologicals). When cells reached a confluency of 80–90%, they were transfected with 1 μg cDNA using Lipofectamine 2000 (Life Technologies). Control cells were transfected with empty vector. After four hours, the cells were trypsinized, plated into 24-well poly-D-lysine coated plates at a density of 1 × 10^5^ cells per well, and incubated for an additional 20–24 hours. The average total protein content was determined for each well of the 24-well plate via bicinchoninic acid using the Pierce BCA Protein Assay Reagent kit^72^.

### Steady-state kinetics of [^3^H]5-HT transport

Flux assays were performed similar to a protocol previously described^73^. Briefly, cells were washed with a modified Ringer’s solution (pH 7.4) containing 5 mM Tris, 7.5 mM HEPES, 120 mM NaCl, 5.4 mM KCl, 1.2 mM CaCl_2_, 1.2 mM MgSO_4_ and then pre-incubated at room temperature in Ringer’s supplemented with 10 mM glucose, 100 μM pargyline, and 100 μM ascorbic acid. Uptake was initiated by first aspirating the well and then adding supplemented Ringer’s containing 0.025–60 μM [^3^H]5-HT (5-hydroxy[^3^H]tryptamine creatine sulfate [specific acitivity 27.8 Ci/mmol, Perkin Elmer, NET498]) to each well and incubating for 5–6 minutes depending on the mutant. For the lowest and highest 5-HT concentrations, preliminary experiments established that transport remained linear for up to 10 min. Reactions were terminated by aspirating the assay solution and washing three times with ice-cold Ringer (1 ml each time). Cells were lysed with 0.5 M NaOH (0.5 ml) and lysate mixed with Opti-Fluor liquid scintillation cocktail (3.5 ml, PerkinElmer) before placing in Beckman liquid scintillation counter. [^3^H]5-HT flux in mock-transfected cells was subtracted from that in SERT-transfected cells to determine specific [^3^H]5-HT transport. To preclude ligand depletion, total uptake counts were always kept at less than 10% counts added. Experiments were performed at least three times, each time triplicate, and the data fit to the Michaelis-Menten equation as implemented in GraphPad Prism 6.

### Paroxetine inhibition of [^3^H]5-HT transport

These experiments were performed as detailed above except that the cells were preincubated for 1–2 hrs at RT with supplemented Ringer’s containing varying concentrations of cold paroxetine (0.03–3000 nM depending on SERT mutant) and the assay buffer contained the appropriate paroxetine concentration as well as 20 nM [^3^H]5-HT, the latter of which was a concentration at least 15-fold lower than the Michaelis constant (*K*_m_) of any mutant. Data in counts/min were normalized to percent of control wells with 0 nM paroxetine. Experiments were performed at least three times, each time in triplicate, with the data fit to a sigmoidal dose-response equation as implemented in GraphPad Prism 6. For each mutant, *IC*_50_s were converted to *K*_i_s with the Cheng-Prusoff equation^74^ using the experimentally-determined *K*_m_ of that mutant. Statistical analysis was carried out using one-way ANOVA and Student’s unpaired t-test (two-tail) in Microsoft EXCEL.

### Preparation of cell membranes for binding assays

Crude membranes were isolated from T-REx 293 cells expressing hsSERT-WT, -A169D, or –A169E. Cells were first transfected with 1 μg cDNA, incubated at 37 °C for 48 hrs, harvested, and washed 3 times with Ringer’s solution. The cells were then sonicated on ice, incubated at −80 °C for 1 hr, thawed, and centrifuged at 15,000 × g for 30 min. The membrane pellet was then resuspended in Ringer’s solution.

### Paroxetine inhibition of [^125^I]RTI-55 binding

Crude membranes (5 μg/ml) were incubated in various concentrations of cold paroxetine (0.003–100 nM depending on SERT mutant) in Ringer’s for 1 hr. Binding was initiated by adding [^125^I]RTI-55 (β-carbomethoxy-3β-[4-iodophenyl]tropane); specific activity 2,200 Ci/mmol, Perkin Elmer, NEX272) to the membrane-paroxetine mix at a final concentration of 0.2 nM and then incubating with gyration in glass tubes for 1 hr at RT. Nonspecific binding was determined by incubating the identical mixes in the presence of 200 μM cold paroxetine. To separate bound from free [^125^I]RTI-55, reactions were vacuum-filtered through a 96-well Multiscreen FB filter plate (MSFBN6B, Millipore) that had been pre-treated with 0.6% polyethyleneimine for 4–16 hrs, and then washed with 6 × 200-μl aliquots of Ringer’s. Filters were transferred to a 96-well Isoplate-96 (Perkin Elmer), scintillation fluid added, and counted in a Packard MicroBeta scintillation counter. To avoid ligand depletion, total binding counts was always less than 10% counts added. Experiments were performed at least 3 times, each time in triplicate, and data fit to a sigmoidal dose-response curve, as implemented in GraphPad Prism 6. Student’s unpaired t-test was used for statistical comparison.

## Additional Information

**How to cite this article**: Davis, B. A. *et al.* Mechanism of Paroxetine (Paxil) Inhibition of the Serotonin Transporter. *Sci. Rep.* **6**, 23789; doi: 10.1038/srep23789 (2016).

## Supplementary Material

Supplementary Information

## Figures and Tables

**Table 1:** Paroxetine Inhibition Constants for SERT Homologues and Mutants^a^.

**hsSERT**	WT	Y95F	**A169D**	**I172M**	A173G	E493N	T497P	V501I
*IC*_50_^b^	2.01 ± 0.36	1.70 ± 0.38	**15.23** ± **2.66**	**4.71 ± 0.54**	0.96 ± 0.16	0.69 ± 0.01	1.71 ± 0.07	2.15 ± 0.05
*K*_i_^c^	1.96 ± 0.35	1.61 ± 0.36	**14.47 ± 2.53**	**4.49 ± 0.52**	0.93 ± 0.15	0.66 ± 0.01	1.60 ± 0.06	2.08 ± 0.05
Fold diff.^d^	1.0	1.2	**−7.4**	**−2.3**	2.1	3.0	1.2	−1.1
n^e^	9	3	9	3	8	4	3	3
*P*^f^			0.0026	0.0128	0.0249	0.0123		

**dmSERT**	WT	F90Y	**D164A**	**M167I**	G168A	N484E	P488T	I492V
*IC*_50_^b^	6.10 ± 1.35	5.31 ± 0.94	**0.86 ± 0.07**	**2.71 ± 0.29**	2.80 ± 0.66	1.90 ± 0.22	20.46 ± 1.86	16.32 ± 1.20
*K*_i_^c^	6.04 ± 1.33	5.26 ± 0.93	**0.86 ± 0.07**	**2.68 ± 0.29**	2.78 ± 0.66	1.83 ± 0.21	20.31 ± 1.85	16.22 ± 1.20
Fold diff.^d^	1.0	1.1	**7.0**	**2.3**	2.2	3.3	−3.4	−2.7
n^e^	9	3	7	4	3	4	4	3
*p*^f^			<0.0001	0.0054	0.0236	0.0101	<0.0001	<0.0001

**ggSERT**	WT		**D209A**	V212I				
*IC*_50_^b^	63.07 ± 6.60		**10.71 ± 1.02**	**24.17 ± 0.41**				
*K*_i_^c^	60.22 ± 6.31		**10.50 ± 1.00**	N.D.				
Fold diff.^d^	1.0		**5.7**	**2.6**				
n^e^	17		8	3				
*p*^f^			<0.0001	0.0023				

^a^Units are expressed in nM and represent the average ± SEM.

^b^Paroxetine concentration at which half of 20 nM [^3^H]5-HT transport is inhibited relative to a “0 nM paroxetine” control.

^c^Inhibition constant calculated from the corresponding *IC*_50_ and SERT mutant Michaelis constant (*K*_m_) via the Cheng-Prusoff equation^74^. Note that the *IC*_50_ and *K*_i_ for the respective SERT construct are approximately the same because the [^3^H]5-HT concentration employed in the inhibition assay was far below the corresponding *K*_m_.

^d^Difference in paroxetine potency between wild-type and mutant SERTs calculated by dividing the corresponding *K*_i_ values, except for ggSERT-V212I, in which case the corresponding *IC*_50_ values were used. Positive and negative numbers refer to gain and loss of paroxetine potency in the mutant, respectively.

^e^Number of individual experiments, each performed in triplicate.

^f^*p* values determined by Students unpaired t-test. Values less than 0.05 indicate a statistically significant difference between the mutant and the WT *K*_i_ or *IC*_50_ values.
