# Supplementary material for: Mechanism of Paroxetine (Paxil) Inhibition of the Serotonin Transporter
Source: Sci Rep. 2016 Apr 1;6:23789. doi: 10.1038/srep23789 (PMC4817154; doi:10.1038/srep23789)
Supplement: Supplementary Information [file srep23789-s1.pdf]

Supplementary Information for

Mechanism of Paroxetine Inhibition of the Serotonin Transporter

Bruce A. Davis[1], Anu Nagarajan[2], Lucy R. Forrest[2], and Satinder K. Singh[1]*

[1]Department of Cellular and Molecular Physiology, Yale University School of Medicine, 333 Cedar Street, New Haven, CT 06520 USA, [2]Computational Structural Biology Section, National Institute of Neurological Disorders and Stroke, 35 Convent Drive, Bethesda, MD 20892 USA

*Correspondence should be addressed to:

Satinder K. Singh, Department of Cellular and Molecular Physiology, Yale University School of Medicine, 333 Cedar Street, New Haven, CT 06520 USA

E-mail: satinder.k.singh@yale.edu

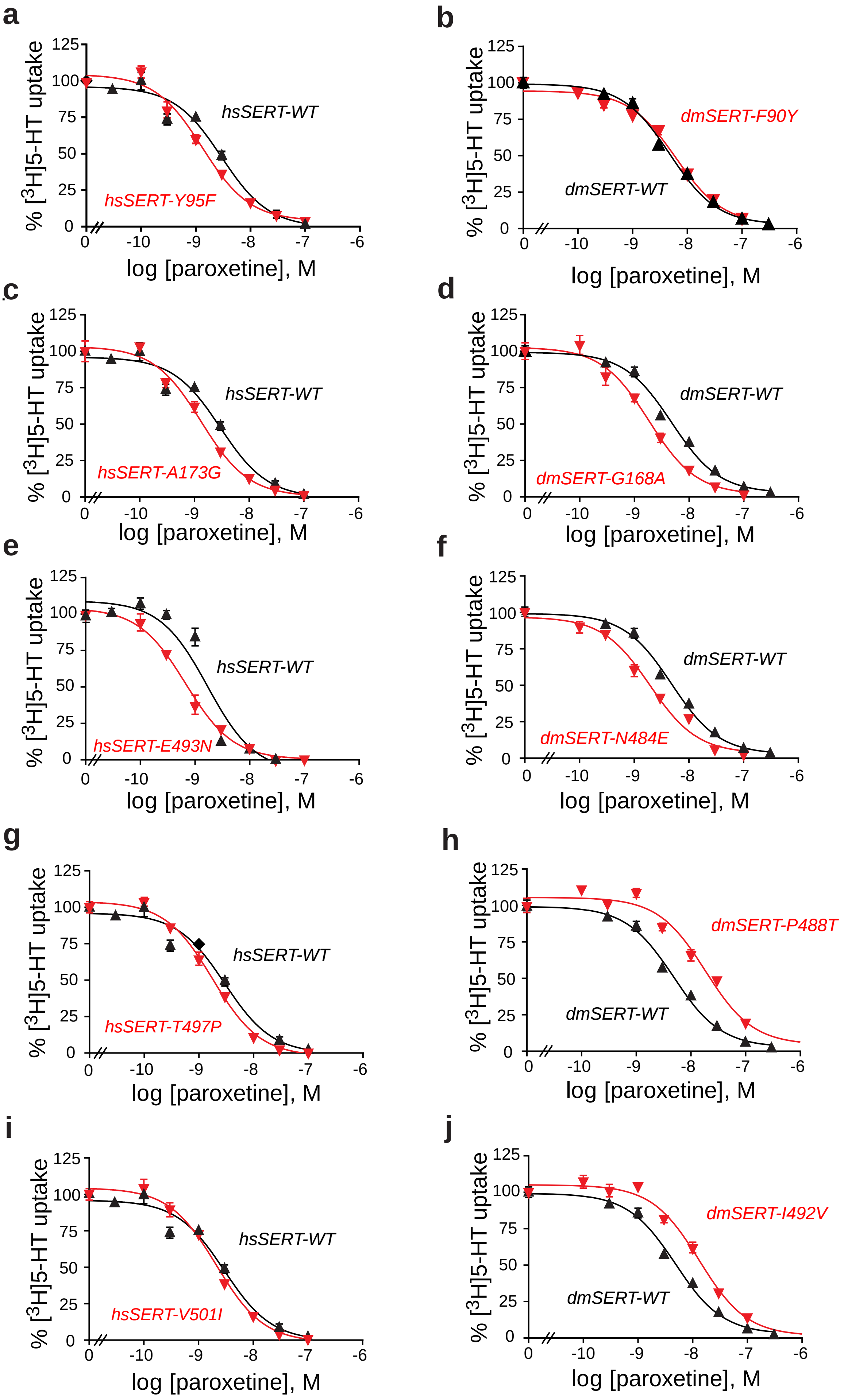

Supplementary Figure S1

Supplementary Figure S1. Paroxetine potencies of hsSERT/dmSERT and corresponding mutants at positions other than A169 and I172 in hsSERT (D164 and M167 in dmSERT) as assessed by inhibition of 20 nM [^{3}H]5-HT transport in transiently-transfected T-REx-293 cells. In all panels, data points and fit for the wild-type (▲) and indicated SERT variant (▼) are shown. (**a**) hsSERT-Y95F, (**b**) dmSERT-F90Y, (**c**) hsSERT-A173G, (**d**) dmSERT-G168A, (**e**) hsSERT-E493N, (**f**) dmSERT-N484E, (**g**) hsSERT-T497P, (**h**) dmSERT-P488T, (**i**) hsSERT-V501I, and (**j**) dmSERT-I492V.

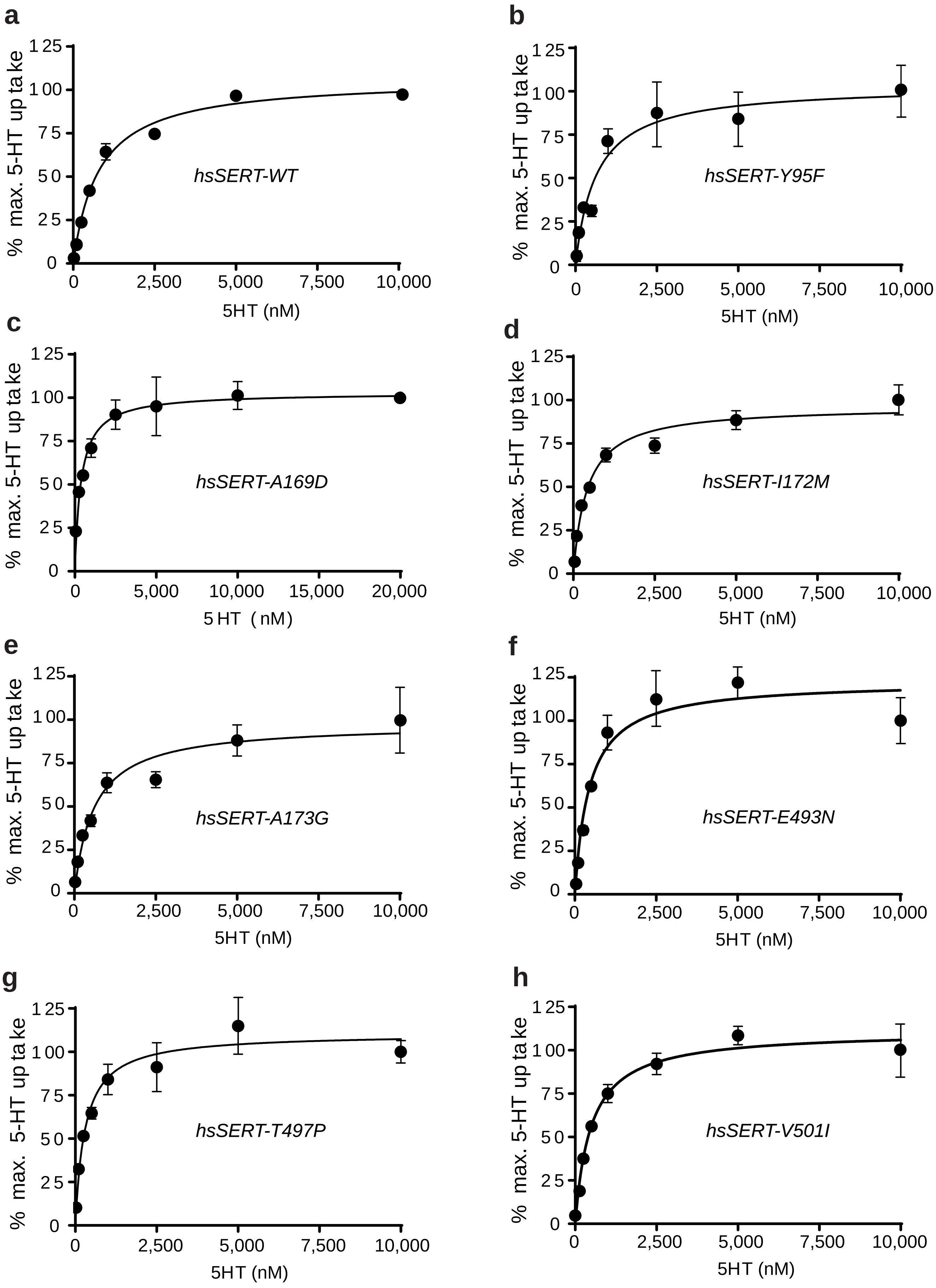

Supplementary Figure S2

Supplementary Figure S2. Steady-state kinetics of [^{3}H]5-HT transport in transiently-transfected T-REx-293 cells expressing hsSERT-WT or the indicated mutant. (**a**) hsSERT-WT, (**b**) hsSERT-Y95F, (**c**) hsSERT-A169D, (**d**) hsSERT-I172M, (**e**) hsSERT-A173G, (**f**) hsSERT-E493N, (**g**) hsSERT-T497P, and (**h**) hsSERT-V501I. For every curve, velocity is expressed as the percent maximal uptake for the indicated hsSERT variant. The graphs are representative of a typical experiment, each of which was performed in triplicate at least three separate times. Each data point (●) is the average of three replicates with the error bars denoting the standard error of the mean (SEM). Data were fit to the Michaelis-Menten equation as implemented in GraphPad Prism 6.

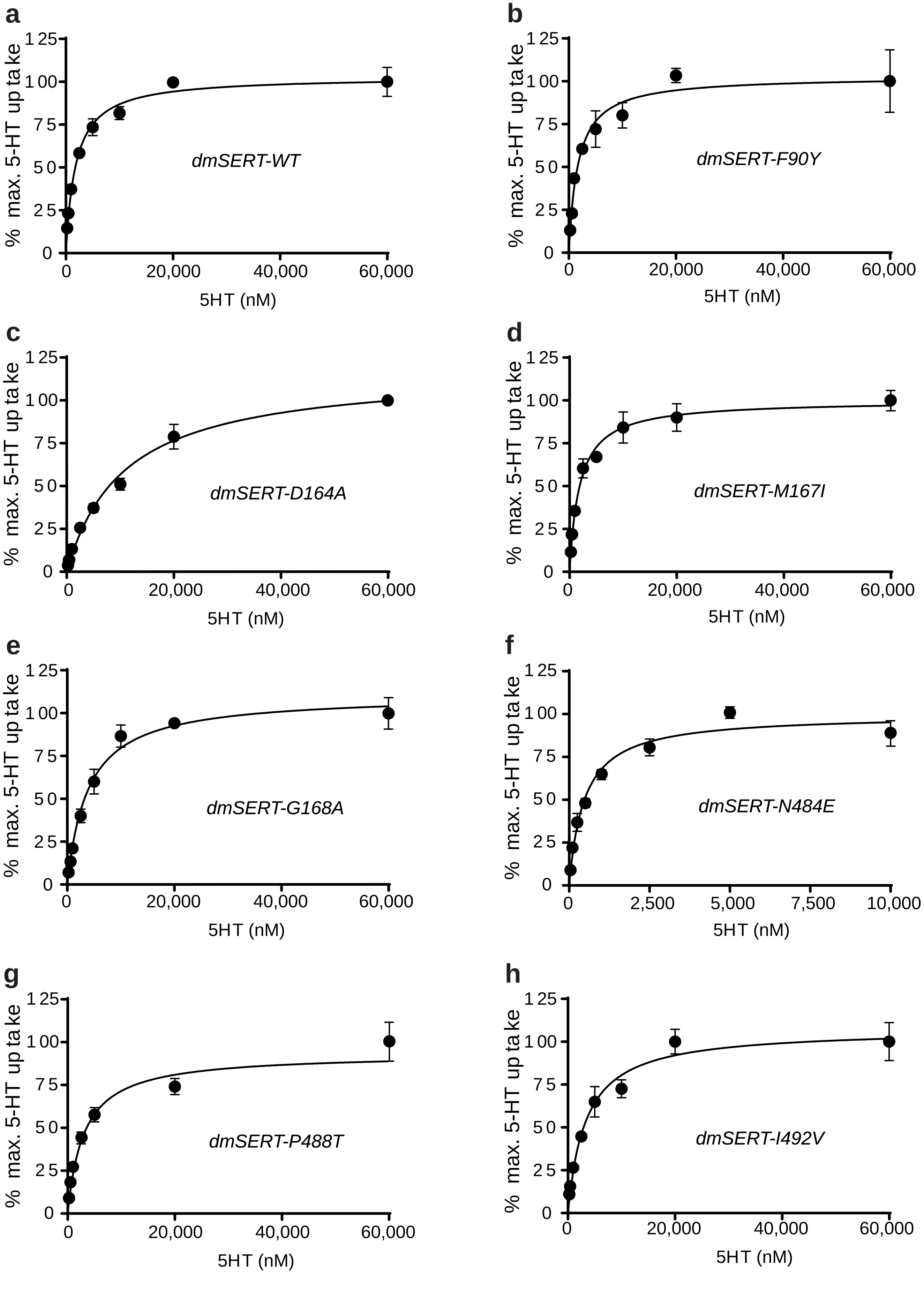

Supplementary Figure S3

Supplementary Figure S3. Steady-state kinetics of [^{3}H]5-HT transport in transiently-transfected T-REx-293 cells expressing dmSERT-WT or the indicated mutant. (**a**) dmSERT-WT (**b**) dmSERT-F90Y (**c**) dmSERT-D164A (**d**) dmSERT-M167I (**e**) dmSERT-G168A (**f**) dmSERT-N484E (**g**) dmSERT-P488T, and (**h**) dmSERT-I492V. For every curve, velocity is expressed as the percent maximal uptake for the indicated dmSERT variant. The graphs are representative of a typical experiment, each of which was performed in triplicate at least three separate times. Each data point (●) is the average of three replicates with the error bars denoting the standard error of the mean (SEM). Data were fit to the Michaelis-Menten equation as implemented in GraphPad Prism 6.

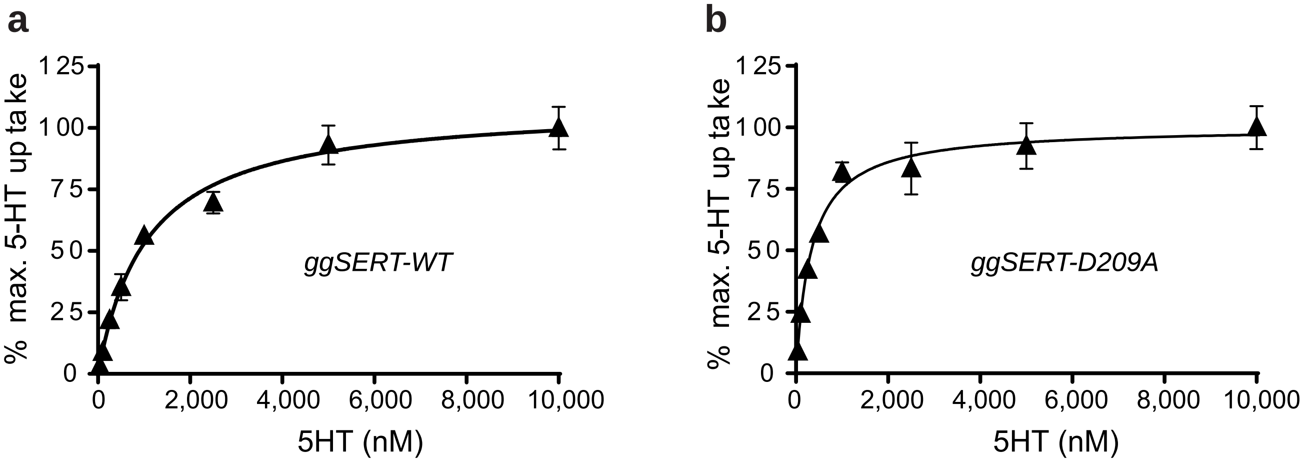

Supplementary Figure S4

Steady-state kinetics of [^{3}H]5-HT transport in transiently-transfected T-REx-293 cells expressing (**a**) ggSERT-WT or (**b**) ggSERT-D209A. For every curve, velocity is expressed as the percent maximal uptake for the indicated ggSERT variant. The graphs are representative of a typical experiment, each of which was performed in triplicate at least three separate times. Each data point (▲) is the average of three replicates with the error bars denoting the standard error of the mean (SEM). Data were fit to the Michaelis-Menten equation as implemented in GraphPad Prism 6.

Supplementary Table S1 | Transport Kinetic Parameters of SERT Homologues & Mutants

	K_m[a]	V_{max}[b]	% wt V_{max}	n[c]
**hsSERT**				
WT	738 ± 34	60.5 ± 6.5	100 ± 10.7	26
Y95F	366 ± 88	23.9 ± 13.9	39.5 ± 22.9	4
A169D	380 ± 62	21.0 ± 3.8	34.7 ± 6.2	7
I172M	417 ± 29	16.8 ± 2.3	27.8 ± 3.7	4
A173G	632 ± 67	38.6 ± 10.3	63.8 ± 17.0	4
E493N	483 ± 121	5.4 ± 2.2	9.0 ± 3.6	3
T497P	308 ± 11	8.9 ± 1.6	14.6 ± 2.7	4
V501I	596 ± 61	35.5 ± 3.0	35.5 ± 3.0	3
**dmSERT**				
WT	1,824 ± 88	64.6 ± 6.9	100.0 ± 10.7	25
F90Y	1,784 ± 121	70.9 ± 2.7	109.7 ± 4.2	4
D164A	10,258 ± 756	87.9 ± 6.8	136.0 ± 10.6	5
M167I	1,487 ± 132	51.6 ± 14.3	79.9 ± 22.1	4
G168A	3,342 ± 272	213.4 ± 39.9	330.3 ± 61.7	4
N484E	522 ± 67	5.2 ± 1.2	8.0 ± 1.8	5
P488T	2,737 ± 277	9.8 ± 0.2	15.1 ± 0.3	4
I492V	3,281 ± 357	58.0 ± 5.7	89.8 ± 8.8	3
**ggSERT**				
WT	423 ± 113	18.4 ± 1.8	100 ± 9.6	3
D209A	971 ± 71	73.8 ± 21.5	218.0 ± 13.5	4
V212I	N.D.[d]	N.D.[d]	N.D.[d]	

[a] Michaelis constant (K_m) units are expressed in nM and represent the average ± SEM (standard error of the mean).

[b] Maximum velocity (V_{max}) units are expressed in pmol/min/mg total cell protein and represent the average ± SEM

[c] Number of individual experiments, each performed in triplicate.

[d] N.D., not determined
